# Correction: Evolution of thorax architecture in ant castes highlights trade-off between flight and ground behaviors

**DOI:** 10.7554/eLife.02322

**Published:** 2014-01-21

**Authors:** Roberto A Keller, Christian Peeters, Patrícia Beldade

Keller RA, Peeters C, Beldade P. 2014. Evolution of thorax architecture in ant castes highlights trade-off between flight and ground behaviors. *eLife* **3**:e01539. doi: http://dx.doi.org/10.7554/eLife.01539. Published 7 January 2014

In the column ‘T1/T2 in queens’ of Table 3 a comma instead of a dot was used for the decimal point in the values for the genera *Aenictus*, *Leptanilla* and *Leptanilloides*.

The article has now been corrected.

